# Complementary feeding practices and associated factors among mothers of children aged 6-23 months in Ethiopia: Secondary data analysis of Ethiopian mini demographic and health survey 2019

**DOI:** 10.1186/s13690-021-00725-x

**Published:** 2021-11-22

**Authors:** Sewunet Sako Shagaro, Be’emnet Tekabe Mulugeta, Temesgen Dileba Kale

**Affiliations:** Department of Health Informatics, School of Public Health, College of Medicine and Health Sciences, Arba Minch University, Arba Minch, Ethiopia

**Keywords:** Complementary feeding practice, Dietary diversity, Meal frequency, Children aged 6–23 months, Ethiopia

## Abstract

**Background:**

Optimal nutrition in early child’s life plays a vital role in improving mental and motor development, reduces the possibility of contracting various infectious diseases and related deaths, decreases the risk of obesity, and fosters better overall development. However, 45% of deaths in children under five years of age that occur globally is attributed to nutrition-related factors and the majority of these deaths occur in low-and middle-income countries. Therefore, this study aims to assess complementary feeding practices and associated factors among mothers of children aged 6–23 months in Ethiopia.

**Method:**

The study used the Ethiopian mini demographic and health survey 2019 data. A two-stage stratified cluster sampling technique was used to select 1465 mothers of children aged 6–23 months in Ethiopia. Two-level multilevel mixed-effects logistic regression model analysis was computed, and variables with *p*-value of less than 5% and an adjusted odds ratio with a 95% confidence interval in the final model were reported as statistically significant factors with appropriate complementary feeding practice.

**Result:**

The overall prevalence of appropriate complementary feeding practice among mothers of children aged 6–23 months was 9.76%. In our study, mothers who attended primary[AOR = 2.72; 95%CI: 1.47–5.01], secondary[AOR = 2.64; 95%CI: 1.18–5.92] and higher school[AOR = 5.39; 95%CI: 2.29–12.64], being from medium income household[AOR = 2.89; 95%CI: 1.41–5.92], attended 1–3 times ANC visits in index pregnancy[AOR = 0.41; 95%CI: 0.18–0.89], mothers who have 12–17 months[AOR = 1.96; 95%CI: 1.16–3.33] and 18–23 months old children[AOR = 2.61; 95%CI: 1.49–4.54], currently breastfeeding mothers[AOR = 3.69; 95%CI: 1.73–7.91], mothers from pastoralist contextual regions[AOR = 0.29; 95%CI: 0.09–0.91], and mothers who have resided in rural areas[AOR = 0.49; 95%CI: 0.25–0.97] were factors significantly associated with appropriate complementary feeding practice.

**Conclusion:**

This study showed low prevalence of appropriate complementary feeding practice. Therefore, the concerned health authorities need to strengthen the existing approaches designed for provision of nutrition education particularly targeting mothers who are unschooled, who have 6–11 months old children, live in pastoralist regions and reside in rural parts of the country, and create strategies that improve maternal job opportunities.

## Background

National and international organizations, including WHO, recommend early initiation of breastfeeding within 1 h of birth and exclusive breastfeeding for the first six months of life [1]. Thereafter, infants should receive complementary foods at 6 months while breastfeeding continues up to two years or beyond. Complementary feeding is defined as the process starting when breast milk alone is no longer sufficient to meet the nutritional requirements of infants and young children (6–23 months), and therefore other foods and liquids are needed, along with breast milk [2, 3].

Complementary foods that are introduced timely, nutritionally adequate, safe; and properly fed together with continued breastfeeding during the first 2 years of a child’s life can save over 820,000 children’s lives every year [4]. However, early commencement of complementary feeding has an adverse effect on children’s adiposity and an increased risk for being overweight [5]. It is also responsible for increasing risk of illness as infants and young children receive less of the protective factors in breast milk, diarrhea, total replacement of breast milk, and increasing the risk of mothers becoming pregnant. Conversely, introducing complementary feeding too late may result in micronutrients deficiencies, energy and nutrient gap, and slow growth [6]. Optimal nutrition in early child’s life plays a vital role in improving mental and motor development, reduces the possibility of contracting various infectious diseases and related deaths, decreases the risk of obesity, and fosters better overall development [7–9]. On the other hand, inappropriate complementary feeding practices may increase risk of malnutrition, deficiencies of nutrients, diarrhea and respiratory tract infections; and slow growth and development of children [10, 11].

However, in most countries less than half of infants and young children 6–23 months of age meet the dietary diversity and feeding frequency that are appropriate to their age [12]. According to EDHS 2016, the prevalence of minimum dietary diversity, minimum meal frequency, and minimum acceptable diet in the country was 14, 45, and 7% respectively [13].

Evidence has shown that 45% of deaths in children under five years of age that occur globally is attributed to nutrition-related factors and the majority of these deaths occur during the first year of life. Low-and middle-income countries are countries where the majority of these deaths occur [2]. Furthermore, 149 million children under 5 were stunted, 45 million were wasted and 38.9 million were overweight or obese out of the total children under 5 in the world during 2020 [4]. Likewise, the 2019 Ethiopia mini demographic and health survey (EMDHS) report shows that 7% of children under 5 years are wasted, 37% are stunted and 21% of all children are underweight [14]. Hence, realizing sustainable socioeconomic development and poverty reduction without improving appropriate feeding practices will be unbearable. As stated in literature, the high morbidity and mortality among infants and young children is attributed mainly by inappropriate infants and young children feeding practices and poor quality of complementary foods [2].

In Ethiopia, the government has designed different nutrition related strategies in its development plans and highlights the role of nutrition in bringing sustainable development. The Seqota Declaration is one of the components of the second National Nutrition Program (NNP II) of Ethiopia which aimed to end child undernutrition by 2030 [15]. However, malnutrition is still widespread in the country and its determinants are multifaceted. Poverty; access to safe, nutritious, and diverse food; access to health care; low education levels; unhealthy environments and hygiene practices; and low levels of awareness about nutrition are among numerous roots of undernutrition in Ethiopia [7, 15–17]. The promotion of appropriate complementary feeding practices decreases the incidence of stunting and results in better health and growth outcome [18].

Independent predictors for appropriate complementary feeding practice from previous similar studies conducted in different places include maternal education, wealth index, child’s age, exposure to media, ANC visit, postnatal checkup, maternal occupation, institutional delivery, and geographical region [19–31].

Few studies in Ethiopia show that complementary feeding practices are inadequate [19, 20, 24]. Also, all of the studies conducted identified only individual-level variables though community-level variables like geographic regions that could affect the practice were not investigated [20, 25, 32–34]. Moreover, nationwide complementary feeding practice was not yet studied using the revised (2021) indicators for assessing infant and young child feeding practices as of the investigators knowledge [35]. Therefore, this study aims to determine the prevalence of appropriate complementary feeding practices and associated factors among mothers of children aged 6 to 23 months in Ethiopia. Ultimately, the findings of this study will help the policy makers, planners and other stakeholders to develop effective strategies and policies concerning complementary feeding practice of infants and young children.

## Methods

### Study design and setting

A community-based cross-sectional study design was conducted in Ethiopia from March, 2019, to June, 2019 among mothers of children aged 6–23 months. Ethiopia is a landlocked country located in the horn of Africa. The country is divided into nine geographical regions namely Tigray, Afar, Amhara, Oromia, Somali, Benishangul-Gumuz, SNNPR, Gambella and Harari; and two administrative cities (Addis Ababa and Dire Dawa).

### Source and study population

The source population of this study was all Ethiopian mothers of children aged 6–23 months. Whereas, the study population of this study was mothers of children 6–23 months of age living in the randomly selected enumeration areas of the country during the year of survey. Accordingly, 1465 mothers of children aged 6–23 months data were extracted from the 2019 EMDHS datasets.

### Inclusion and exclusion criteria

All eligible mothers with children aged 6–23 months and who live in Ethiopia were included in the study. However, mothers who were seriously ill or unable to communicate during the data collection period were excluded from the study.

### Sampling and data source

Central Statistical Agency (CSA) of Ethiopia, in collaboration with its partner has conducted the second Ethiopia mini demographic and health survey (EMDHS) from March 2019 to June 2019. The survey included all the nine regional states and two city administration of Ethiopia. Thus, the data source for the current study is the 2019 EMDHS data.

The demographic and health survey (DHS) often uses the most recent census frame to draw samples for the survey. Hence, the current survey has used enumeration areas (EAs) created for the upcoming Ethiopia population and housing census as a sampling frame. Normally, DHS samples are stratified by geographic region and by urban or rural areas within each region of the country. Initially, within each stratum, EAs are selected by using probability proportional to size (PPS) sampling method. Then, systematic equal probability sampling techniques are used to select a fixed number of households in the selected EAs.

Finally, the 2019 EMDHS survey covered 8663 households out of the selected 8794 households providing a response rate of 98.5%. From the 9012 women identified for the interview around 8885 women completed the interview, yielding a response rate of 98.6% [14]. Further information on the survey sampling strategies are available in the DHS guideline [36].

### Measurement of variables

**Dependent variable:** Appropriate complementary feeding practices was the response variable. It was measured using the following composite indicators recommended by the WHO [35].

- **Introduction of solid, semi-solid or soft foods 6–8 months**: The proportion of infants 6–8 months of age who consumed solid, semi-solid or soft foods during the previous day.
- **Minimum dietary diversity 6–23 months**: The proportion of children 6–23 months of age who consumed foods and beverages from at least five out of eight defined food groups during the previous day. The eight food groups are breast milk; grains, roots, tubers, and plantains; pulses (beans, peas, lentils), nuts and seeds; dairy products (milk, infant formula, yogurt, cheese); flesh foods (meat, fish, poultry, organ meats); eggs; vitamin-A rich fruits and vegetables; and other fruits and vegetables.
- **Minimum meal frequency 6–23 months**: The proportion of children 6–23 months of age who consumed solid, semi-solid or soft foods (but also including milk feeds for non-breastfed children) at least the minimum number of times during the previous day. The minimum number of times is defined as two feedings of solid, semi-solid or soft foods for breastfed infants aged 6–8 months; or three feedings of solid, semi-solid or soft foods for breastfed children aged 9–23 months; or four feedings of solid, semi-solid or soft foods or milk feeds for non-breastfed children aged 6–23 months whereby at least one of the four feeds must be a solid, semi-solid or soft feed.
- **Minimum acceptable diet 6–23 months**: The proportion of children 6–23 months of age who consumed a minimum acceptable diet during the previous day. The minimum acceptable diet is defined as for breastfed children: receiving at least the minimum dietary diversity and minimum meal frequency for their age during the previous day or for non-breastfed children: receiving at least the minimum dietary diversity and minimum meal frequency for their age during the previous day as well as at least two milk feeds.
- **Appropriate complementary feeding practices**: Infants and young children feeding practices that satisfies the minimum dietary diversity, minimum meal frequency and introduction of solid, semi-solid or soft foods at the recommended diversity, frequency and time of WHO.
- **Inappropriate complementary feeding practices**: Infants and young children feeding practices that did not satisfy one of the above three criteria of WHO.

**Individual-level independent variables**: this level includes maternal socio-demographic factors, maternal health service and related factors, and child related factors of the study.

i. **Socio-demographic factors** such as maternal age, maternal education, marital status, religion, family size, owned radio/television, wealth index
ii. **Maternal health service and related factors** such as parity, ANC visits attended in index pregnancy, place of delivery, current breastfeeding status, PNC checkup,
iii. **Child related factors** such as sex of the child, age of the child, birth order, preceding birth interval, and number of children 5 and under in household.

**Community-level independent variables**: residence and contextual regions were community-level factors of this study (Table 1).

**Table 1:** Description of individual and community-level variables of mothers of children aged 6–23 months in Ethiopia, 2019

Variables	Description
Maternal age	It is the current age of women recoded as 15–24, 25–34, and 35–49.
Maternal educational status	This is the level of education a woman attained and recoded as no education, primary, secondary and higher.
Religion	This variable is the religious group to which the woman associates herself and recoded to dominant religious groups: Muslim, Orthodox, Protestant, and others (Catholic, traditional and others).
Family size	It is the total number of household members and recoded as ≤ 5 or > 5 members.
Wealth index	In DHS the wealth index is calculated using data on a household’s ownership of selected assets. Each household asset is assigned a weight score generated through PCA. The resulting asset scores are standardized and summed by household and individuals are ranked according to the total score of the household in which they reside. Consequently, it is grouped as poorest, poorer, middle, richer and richest. Finally, we have re-categorized the variable as Low income (poorest and poorer), Middle income and High income (richer and richest).
Region	Region of residence is typically the first administrative level within the country, or a grouping of the first administrative level. There are 11 regions in Ethiopia which were categorized into three contextual regions in our study. These are agrarian regions (Tigray, Amhara, Oromia, Benishanul-Gumuz, SNNPR, Gambela and Harari), pastoralist regions (Afar and Somali) and city administrations (Addis Ababa and Dire Dawa).
Place of residence	It is the designation of the cluster or enumeration area as an urban area or a rural area.

### Data processing and analysis

Data extracted from the 2019 EMDHS was cleaned, re-coded and analyzed using STATA/SE version 14.0 statistical software packages. Sample weight was applied to manage for sampling errors and non-responses. Descriptive statistics were used to summarize both the individual and community-level variables. DHS data were organized hierarchically, children are nested within households and households were nested within clusters. This clearly indicates that children from the same clusters are more similar than those from different cluster in terms of the outcome of interest. Subsequently, this violates the independence of observation assumption of traditional logistic regression. To overcome such difficulties and to predict fixed effects of the explanatory variables and community-level random effects on the dependent variable we used a two-level multilevel mixed-effects logistic regression model. Intra-class correlation coefficient (ICC) and proportional change in deviance (PCV) were checked to quantify the magnitude of the clustering effect and the degree to which community-level factors explain the unexplained variance of the null model. In this study, a model with the lowest deviance was used as the best-fitted model. Accordingly, variables with *p*-value < 0.25 in the bivariable analysis were included in the multivariable multilevel mixed-effects logistic analysis. In the multivariable analysis, those variables with p-value less than 5% and adjusted odds ratio (AOR) with 95% confidence interval (CI) were reported as statistically significant variables with appropriate complementary feeding practices. Existence of multicollinearity between covariates was checked by using variance inflation factor (VIF) and the mean VIF was found to be 2.25, indicating absence of significant collinearity among explanatory variables.

## Result

### Characteristics of the study participants

Among 1465 participants included in the analysis, 650 (44.45%) had no formal education, half (49.56%) were in the age range of 25–34, and the majority (95.33%) were currently married. Out of the total women, nearly one third (36.82%) were Muslim religion followers, more than half (55.01%) have family size greater than 5 members and almost one fifth (18.83%) were in medium wealth class. Regarding obstetric and use of health services, half (49.80%) were multiparous, nearly one quarter (23.62%) had no ANC visit, more than half (55.09%) gave birth at health facility and only one tenth (10.24%) had PNC checkup. Out of the infants and young children included in the study, one third (32.55%) were 6–11 months old, almost half (52.22%) were male, and nearly one quarter (24.18%) were first-born. Of the participants, tenth (10.09%) were from households which had 1–2 numbers of under 5 children, the majority (88.64%) were from agrarian regions and nearly three fourth (71.77%) resided in rural areas (Table 2).

**Table 2:** Characteristics of mothers of children aged 6–23 months in Ethiopia, 2019

Variables	Category	Weighted frequency (N)	Percent (%)
Maternal education	No education	650.09	44.45
	Primary	609.22	41.65
	Secondary	119.73	8.19
	Higher	83.56	5.71
Respondent’s current age	15–24	472.64	32.31
	25–34	724.94	49.56
	35–49	265.03	18.12
Marital status	Never in union	5.82	0.40
	Currently married	1394.30	95.33
	Formerly married	62.48	4.27
Religion	Muslim	474.20	32.42
	Orthodox	538.54	36.82
	Protestant	412.70	28.22
	Others	37.16	2.54
Family size	≤5	804.51	55.01
	> 5	658.11	44.99
Owned radio or television	No	943.80	64.53
	Yes	518.80	35.47
Wealth index	Low income	603.90	41.29
	Medium income	275.42	18.83
	High income	583.30	39.88
Parity	Primiparous	349.63	23.90
	Multiparous	728.38	49.80
	Grand multiparous	384.59	26.30
ANC visits attended in index pregnancy	None	345.50	23.62
	1–3	450.67	30.81
	4 or more visits	666.43	45.56
Place of delivery	Health facility	805.76	55.09
	Home	656.85	44.91
PNC checkup	No	1311.73	89.76
	Yes	149.61	10.24
Current age of child in months	6–11	476.07	32.55
	12–17	551.31	37.69
	18–23	435.23	29.76
Sex of child	Male	763.83	52.22
	Female	698.77	47.78
Birth order	First-born	353.60	24.18
	Second-fourth	691.05	47.25
	Fifth or more	417.96	28.58
Preceding birth interval	< 24 months	222.96	15.24
	≥ 24 months	1239.64	84.76
Number of under 5 children in household	No child	5.71	0.39
	1–2	1309.27	89.52
	3 and above	147.62	10.09
Currently breastfeeding	No	216.51	14.80
	Yes	1246.11	85.20
Region	Agrarian	1296.52	88.64
	Pastoralist	108.92	7.45
	City administration	57.17	3.91
Place of residence	Urban	412.92	28.23
	Rural	1049.69	71.77

### Prevalence of appropriate complementary feeding practices

**Fig. 1:**
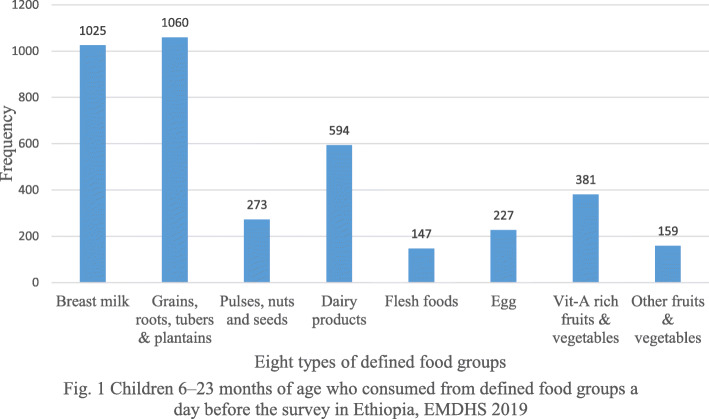
Children 6–23 months of age who consumed from defined food groups a day before the survey in Ethiopia, EMDHS 2019

Majority (83.28%) of infants (6–8 months) enrolled in the study consumed solid, semi-solid or soft foods (SSSF) a day before the interview (Fig. 1).

**Fig. 2:**
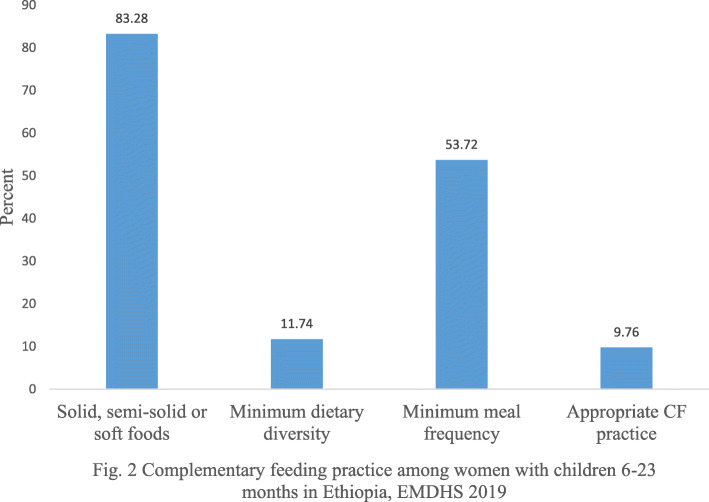
Complementary feeding practice among women with children 6-23 months in Ethiopia, EMDHS 2019

Out of the total infants and young children (6–23 months) included in the study, relatively one tenth (11.74%) of infants and young children consumed foods and beverages from at least five out of eight defined food groups during the previous day of the survey. Of these infants and young children, more than half (53.72%) consumed solid, semi-solid or soft foods at least the minimum number of times during the day preceding the survey. Successively, the overall prevalence of appropriate complementary feeding practices among mothers of children aged 6–23 months in Ethiopia was 9.76% (95% CI: 8.24–11.28%) (Fig. 2).

### Factors associated with appropriate complementary feeding practices

In the bivariable analysis, maternal education, family size, religion, possession of radio or television, wealth index, parity, ANC visits attended in index pregnancy, place of delivery, PNC checkup, age of child, current breastfeeding status, birth order, under 5 children in the household, contextual region; and place of residence were found to be associated with complementary feeding practices. All these variables having *p*-value of less than 0.25 in the bivariate analysis were re-entered into multivariable analysis. The result of multivariable analysis showed that maternal education, wealth index, ANC visits attended in index pregnancy, age of child, current breastfeeding status, contextual region and place of residence were significantly associated with appropriate complementary feeding practices. Women who attended primary, secondary and higher school were 2.7 [AOR = 2.72; 95%CI: 1.47–5.01], 2.6 [AOR = 2.64; 95%CI: 1.18–5.92], and 5.4 [AOR = 5.39; 95%CI: 2.29–12.64] times more likely practice appropriate complementary feeding compared with those women who had no formal education. The odds of appropriate complementary feeding practices was 2.9 [AOR = 2.89; 95%CI: 1.41–5.92] times more likely to be practiced among women from medium income households as compared to women from low income households. The odds of appropriate complementary feeding practices was 59% [AOR = 0.41; 95%CI: 0.18–0.89] lower among women who had attended 1–3 times ANC visits in index pregnancy as compared to those women who had no visits. Compared to those women who had 6–11 months old children, women who had 12–17 months and 18–23 months old children were 1.9[AOR = 1.96; 95%CI: 1.16–3.33] and 2.6[AOR = 2.61; 95%CI: 1.49–4.54] times more likely practice appropriate complementary feeding. The odds of appropriate complementary feeding practices was 3.7[AOR = 3.69; 95%CI: 1.73–7.91] times higher among women who were currently breastfeeding as compared to their counterparts. Among community-level factors, women from pastoralist contextual regions were 71% [AOR = 0.29; 95%CI: 0.09–0.91] less likely to practice appropriate complementary feeding as compared to women from agrarian contextual regions. Moreover, the odds of appropriate complementary feeding practice was 51% [AOR = 0.49; 95%CI: 0.25–0.97] lower among women who have resided in rural areas as compared to their counterparts (Table 3).

**Table 3:** Multivariable multilevel analysis of factors associated with appropriate complementary feeding practices among mothers of children age 6–23 months in Ethiopia, 2019

Variables	Null model	Model I	Model II	Model III
**Maternal education**				
No education		1		1
Primary		2.83[1.55–5.17]**		2.72[1.47–5.01]**
Secondary		2.74[1.24–6.06]*		2.64[1.18–5.92]*
Higher		6.07[2.63–13.96]***		5.39[2.29–12.64]***
**Religion**				
Muslim		1		1
Orthodox		1.41[0.85–2.36]		1.21[0.71–2.06]
Protestant		0.69[0.35–1.34]		0.59[0.29–1.19]
Other		0.34[0.04–3.32]		0.31[0.03–2.96]
**Family size**				
≤5		1		1
> 5		1.05[0.64–1.71]		1.04[0.63–1.72]
**Owned radio or television**				
No		1		1
Yes		1.51[0.91–2.52]		1.33[0.78–2.27]
**Wealth index**				
Low income		1		1
Medium income		3.03[1.49–6.18]		2.89[1.41–5.92]**
High income		2.70[1.32–5.53]		2.01[0.93–4.32]
**Parity**				
Primiparous		1		1
Multiparous		1.83[0.46–7.33]		1.83[0.44–7.67]
Grand multiparous		2.28[0.28–18.48]		2.57[0.30–21.63]
ANC visits attended in index pregnancy				
None		1		1
1–3		0.42[0.19–0.94]*		0.41[0.18–0.89]*
4 or more visits		0.84[0.39–1.81]		0.77[0.36–1.67]
**Place of delivery**				
Health facility		1		1
Home		0.77[0.41–1.46]		0.88[0.46–1.69]
**PNC checkup**				
No		1		1
Yes		1.61[0.93–2.82]		1.57[0.88–2.75]
**Age of child in months**				
6–11		1		1
12–17		1.93[1.14–3.25]*		1.96[1.16–3.33]*
18–23		2.53[1.46–4.38]**		2.61[1.49–4.54]**
**Birth order**				
First-born		1		1
Second-fourth		0.36[0.09–1.43]		0.36[0.09–1.48]
Fifth or more		0.27[0.03–2.13]		0.24[0.03–1.99]
**Under 5 children in the house**				
No child		1		1
1–2		0.92[0.12–7.13]		0.91[0.12–7.12]
3 and above		0.89[0.10–7.92]		0.94[0.10–8.38]
**Currently breastfeeding**				
No		1		1
Yes		3.54[1.70–7.38]**		3.69[1.73–7.91]**
**Contextual region**				
Agrarian			1	1
Pastoralist			0.12[0.04–0.36]***	0.29[0.09–0.91]*
City administration			1.23[0.61–2.48]	1.06[0.54–2.09]
**Type of residence**				
Urban			1	1
Rural			0.21[0.11–0.39]***	0.49[0.25–0.97]*

**Random effect analysis**: As depicted in Table 4, the presence of significant variations of appropriate complementary feeding practices between clusters was supported by the ICC in the empty model (null model). Nearly 43% (ICC in null model) of the variation in appropriate complementary feeding practices among mothers of children aged 6–23 months was due to the variation between clusters. Moreover, the smallest value of deviance observed in the last model implies that model-3 (final model) was the best explanatory model able to explain the variation in appropriate complementary feeding practices between the clusters (Table 4).

**Table 4:** Model comparison for identifying factors affecting appropriate complementary feeding practices among mothers/caregivers of children age 6–23 months in Ethiopia, 2019

Parameters	Null model	Model I	Model II	Model III
**Random effect**				
Community-level variance (SE)	0.69***	0.37**	0.48***	0.40**
ICC (%)	42.68%	18.33%	31.03%	20.38%
PCV	Reference	69.79	39.59%	65.71
AIC	893.79	796.05	839.56	791.88
BIC	904.37	922.95	866.01	934.64
**Model fitness**				
Log likelihood	− 444.896	− 374.02	− 414.78	− 368.94
Deviance	889.78	748.04	829.56	737.88

## Discussion

The study revealed that the prevalence of appropriate complementary feeding practices among women with children 6–23 months in Ethiopia was 9.76% (95% CI: 8.24–11.28%). This finding is in agreement with study findings of 8.5 and 9.5% in Southern Ethiopia [20, 25]. The level of appropriate complementary feeding practice in this study was lower than findings of Northeast Ethiopia (57.7, 56.5, 43%) [26, 27, 31], Northwest Ethiopia (37.2%) [28] and Northern Ghana (15.7%) [29]. But the current prevalence was higher than when compared to the findings from Southwestern Nigeria [30] which was 4.2%. The reasons for the relatively lower proportion might be as a result of sample size difference and study population. Our study covered all the regions of the country which is composed of different communities but the previous studies covered a single town or district population. Additionally, the variation might be attributed to socioeconomic status and cultural differences observed among intra-country and inter-country.

In the present study eight out of ten (83.3%) infants of 6–8 months old consumed solid, semi-solid or soft foods a day before the survey. The finding of this study satisfies the WHO recommendation (> 80%) of initiation of complementary feeding for 6–8 months old infants. Our study findings are higher than similar studies conducted in Northeast Ethiopia (56.2%) [31] and from the findings of secondary data analysis of Ethiopia demographic and health survey (59.5%) [21]. This might be due to differences in the study period, maternal education level and increased involvement of health extension workers. The government of Ethiopia has trained and deployed more than 42,000 health extension workers in the country whose main roles are providing health education, preventing diseases and delivering basic curative services [37].

The proportion of minimum dietary diversity in our study was 11.74%. This result is lower than the findings from the studies done in different parts of Ethiopia [34, 38, 39], Sub-Saharan Africa [29, 40, 41] and Asia [23, 42]. This difference could be attributed to variation in the measurement of minimum dietary diversity. Previous studies measured minimum dietary diversity of children 6–23 months of age who consumed foods and beverages from at least four out of seven defined food groups while we used at least five out of eight defined food groups during the previous day. Hence, the use of four out of seven defined food groups may overestimate the prevalence. In addition, the variation might be due to differences in cultural and socio-economic status of the study population.

The result of this study revealed that the proportion of children who consumed solid, semi-solid or soft foods at least the minimum number of times (minimum meal frequency) during the prior day of the survey was found to be 53.72%. This figure is lower than findings from studies conducted in Ethiopia [25, 39, 43], Northern Tanzania [41], and Northern Ghana [29]. However, the current study finding is higher than studies conducted in Ethiopia (33.8, 47.1%) [31], [20], Sub-Saharan Africa (29.8, 41.9%) [40, 44] and Pakistan 38% [23]. The possible reasons for the variation between studies might be as a result of differences in household’s income status, which determines the availability and quality of complementary food items for the infant and child. Also, maternal educational differences might be another possible reason for the dissimilarity among the findings of the studies.

Maternal education was significantly associated with appropriate complementary feeding practice. The odds of appropriate complementary feeding practice were lesser among mothers who had no formal education than mothers who attended primary, secondary and above school. This finding is consistent with similar studies conducted in different places [25, 27, 28, 45–49]. The possible explanation could be mothers who attended formal education have more exposure to media, and maternal and child health services. Hence, they could have good knowledge about the appropriate complementary feeding practices so that they could be able to integrate knowledge obtained from Medias and by visiting health institutions [28, 47]. Moreover, mothers who completed primary or above schools have a high probability of securing job opportunities. This in turn will increase the income level of the household and as a result they might have confidence and capability to improve their infant and child feeding practice.

Furthermore, the increased odds of appropriate complementary feeding practices were found among mothers from medium income households as compared to mothers from low income households. This result is concordant with previous similar studies done in Southern Ethiopia, Tanzania and Pakistan [23, 50, 51]. Because of the fact that, a household’s income level highly determines the availability of food items in the households. In our study nearly half of the mothers were from households of low income status. Hence, they cannot afford and are able to regularly secure defined food groups for their infants and young children.

In our study, the age of infants and young children was positively associated with appropriate complementary feeding practice. Women who had 12–17 months and 18–23 months old children were more likely to practice appropriate complementary feeding than women who had 6–11 months old children. This shows that appropriate complementary feeding practice of mothers increases as the age of children increases. In the present study, appropriate complementary feeding practices of mothers of children aged 6–11 months was 6.6% while it was 10.6 and 12.5% among mothers of children aged 12–17 months and 18–23 months respectively. This finding was in agreement with those studies conducted in Southwest Ethiopia [39], Southern Ethiopia [25, 49], Western Ethiopia [52] and Tanzania [50].

The present study revealed that mothers from pastoralist regions practices appropriate complementary feeding lower than mothers from agrarian regions of Ethiopia. This is consistent with similar studies conducted elsewhere [23, 50]. The possible explanation for the regional differences could be due to variation in agro-ecological characteristics and cultures of the people. Similarly, the study reported that the odds of appropriate complementary feeding practices among mothers who lived in rural settlements was lower than mothers who lived in urban areas.

The main strengths of this study include the use of a multilevel mixed-effects model that can overcome the problem of hierarchical nature of the DHS data. Additionally, we have used the most recent (the 2019 EMDHS) and a nationwide survey data that help the concerned health authorities both at central and local levels to set appropriate intervention strategies. Also, the study determined the prevalence of appropriate complementary feeding practices based on the revised WHO indicators (WHO 2021) for assessing infant and young child feeding practices.

Our study is not free from limitations. Recall and social desirability bias may be introduced while estimating the exact meal frequency, types of food items and consumption of complementary food as it depends on the response of mothers self-report. The study also failed to assess appropriate complementary feeding practices with other relevant variables such as exposure to media, maternal occupation, and women autonomy over household’s earnings and power over household’s purchases due to absence of the data in the current mini DHS datasets.

## Conclusion

This study showed low prevalence of appropriate complementary feeding practice. Therefore, the concerned health authorities need to strengthen the existing approaches designed for provision of nutrition education particularly targeting mothers who are unschooled, who have 6–11 months old children, live in pastoralist regions and reside in rural parts of the country, and create strategies that improve maternal job opportunities.

## Data Availability

The survey dataset used in this analysis is publicly available at http://www.dhsprogram.com/data/.
